# Supplementary material for: Causal Relationship between Aging and Anorexia Nervosa: A White-Matter-Microstructure-Mediated Mendelian Randomization Analysis
Source: Biomedicines. 2024 Aug 16;12(8):1874. doi: 10.3390/biomedicines12081874 (PMC11351342; doi:10.3390/biomedicines12081874)
Supplement: Supplementary file 1 [file biomedicines-12-01874-s001.zip › Supplementary Figure/Figure S4/Singe SNP plot of MR analysis for TL to ubm-b-1607.pdf]

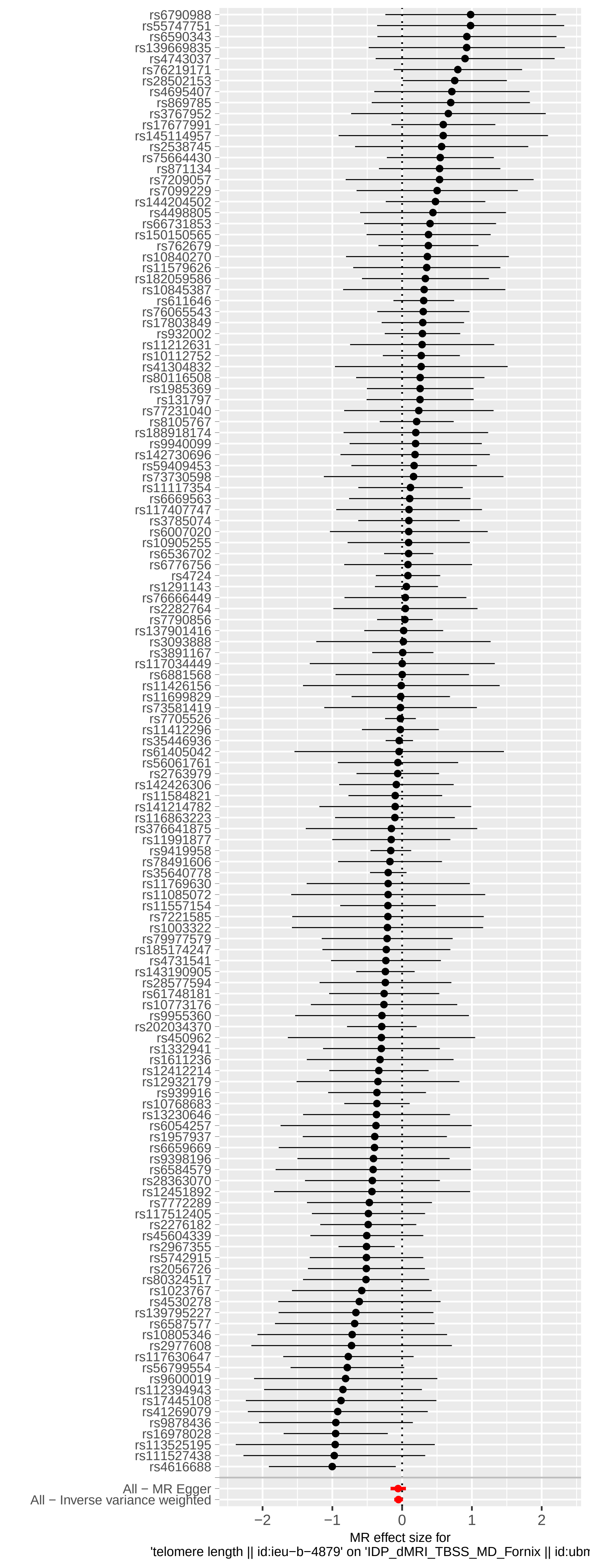
rs6790988
rs55747751
rs6590343
rs139669835
rs4743037
rs76219171
rs28502153
rs4695407
rs869785
rs3767952
rs17677991
rs145114957
rs2538745
rs75664430
rs871134
rs7209057
rs7099229
rs144204502
rs4498805
rs66731853
rs150150565
rs762679
rs10840270
rs11579626
rs182059586
rs10845387
rs611646
rs76065543
rs17803849
rs932002
rs11212631
rs10112752
rs41304832
rs80116508
rs1985369
rs131797
rs77231040
rs8105767
rs188918174
rs9940099
rs142730696
rs59409453
rs73730598
rs11117354
rs6669563
rs117407747
rs3785074
rs6007020
rs10905255
rs6536702
rs6776756
rs4724
rs1291143
rs76666449
rs2282764
rs7790856
rs137901416
rs3093888
rs3891167
rs117034449
rs6881568
rs11426156
rs11699829
rs73581419
rs7705526
rs11412296
rs35446936
rs61405042
rs56061761
rs2763979
rs142426306
rs11584821
rs141214782
rs116863223
rs376641875
rs11991877
rs9419958
rs78491606
rs35640778
rs11769630
rs11085072
rs11557154
rs7221585
rs1003322
rs79977579
rs185174247
rs4731541
rs143190905
rs28577594
rs61748181
rs10773176
rs9955360
rs202034370
rs450962
rs1332941
rs1611236
rs12412214
rs12932179
rs939916
rs10768683
rs13230646
rs6054257
rs1957937
rs6659669
rs9398196
rs6584579
rs28363070
rs12451892
rs7772289
rs117512405
rs2276182
rs45604339
rs2967355
rs5742915
rs2056726
rs80324517
rs1023767
rs4530278
rs139795227
rs6587577
rs10805346
rs2977608
rs117630647
rs56799554
rs9600019
rs112394943
rs17445108
rs41269079
rs9878436
rs16978028
rs113525195
rs111527438
rs4616688
All − MR Egger
All − Inverse variance weighted
−2
−1
0
1
2
MR effect size for
'telomere length || id:ieu−b−4879' on 'IDP_dMRI_TBSS_MD_Fornix || id:ubm
